# Nutrition assessment and management in children on peritoneal dialysis

**DOI:** 10.1007/s00467-007-0719-4

**Published:** 2009-04-01

**Authors:** Fabio Paglialonga, Alberto Edefonti

**Affiliations:** Pediatric Nephrology and Dialysis Unit, Clinica Pediatrica G. e D. D Marchi, Fondazione IRCCS Ospedale Maggiore Policlinico, Mangiagalli e Regina Elena, Via Commenda, 9, Milan, 20122 Italy

**Keywords:** Nutrition, Malnutrition, Nutritional status, Peritoneal dialysis, Cachexia, Anthropometry, Bioimpedance analysis

## Abstract

Protein-calorie malnutrition, otherwise known as cachexia, is a common problem in children undergoing chronic peritoneal dialysis (PD) and is a frequent source of significant morbidity and mortality. Recent evidence suggests that the main factors involved in the pathogenesis are metabolic acidosis, a decreased response to anabolic hormones, and chronic inflammation, associated with hormonal imbalances and an increased metabolic rate. Given the complexity and multifactorial nature of cachexia, the assessment of nutritional status in children on PD requires a complete history and physical examination; assessment of dietary intake, biochemical indices, and anthropometry; and possibly bioimpedance analysis and combined score systems. Its management should likewise be multidisciplinary and include ensuring an adequate energy and protein intake; optimal metabolic control, with the correction of acidosis, anaemia, and hyperparathyroidism; an optimal (or at least adequate) dialysis dose; and, if necessary, prescription of specific drugs such as recombinant human growth hormone.

## Introduction

Protein-calorie malnutrition is common in children undergoing chronic peritoneal dialysis (PD) and is associated with increased morbidity and mortality [1–6]. Although many articles have been published, only a few evidence-based data are available for the paediatric PD population: the many issues still being debated include the best way of assessing nutritional status, the criteria for a diagnosis of cachexia, and the best available measures for preventing and treating it.

The aim of this article is to discuss the most important questions concerning the assessment and management of nutrition in children on PD.

## Definition

An adequate nutritional status can be defined as the maintenance of a normal body composition and a normal pattern of growth, whereas protein-calorie malnutrition can be identified as the loss of muscle mass and protein stores. However, there is still no agreement as to terminology: malnutrition generally describes a condition due to inadequate intake of nutrients in which the metabolic rate is usually reduced, fats are lost, and lean body mass initially preserved. Furthermore, and most importantly, these abnormalities can be reversed by dietary supplements.

Unfortunately, this is not the case for patients with chronic kidney disease (CKD). So, given the multifactorial nature of this condition, some authors have proposed to use the term cachexia to describe the typical abnormalities in the nutritional status of this population. This is a condition characterised by loss of lean body mass combined with normal or even increased fat mass, high resting energy expenditure and inadequate response to nutrient supplementation [7–11].

However, in clinical practice, the term cachexia has so far been restricted to severely malnourished patients and, in the literature, most articles still use the term malnutrition. In this article, preference is given to the term cachexia, and the term malnutrition is used only where aspects of altered nutrient intake are concerned.

## Causes of cachexia in children on PD

The pathogenesis of cachexia in patients on dialysis is complex and multifactorial, as can be seen from the following causes:

I Decreased nutrient intake
  1. Uremic toxins
  2. Abnormalities in hormones controlling energy homeostasis (↑leptin, ghrelin)
  3. Inadequate release and function of neurotransmitters (↓neuropeptide Y, agouti-related peptide)
  4. Resistance to anabolic hormones [insulin, insulin-like growth factor-1 (IGF-1)]
  5. Metabolic acidosis
  6. Systemic inflammation [↑ interleukin (IL)-1, IL-8, tumour necrosis factor-α (TNF-α)]
  7. Inadequate diet and economic/psychosocial problems
  8. Medications affecting food intake
  9. Inadequate dialysis dose/decreased residual renal function
  10. Sense of fullness due to abdominal fluid content
II Increased nutrient losses
  1. Dialysate protein and amino acid losses
  2. Recurrent episodes of peritonitis
  3. Urinary protein losses (dependent on primary renal disease)
III Increase nutritional needs
  1. Metabolic acidosis
  2. Physical inactivity
  3. Intercurrent diseases
  4. Catch-up growth
  5. Inflammatory status

A detailed analysis of all of these factors is beyond the scope of this article, but some issues deserve special mention. Anorexia is very common in patients with end-stage renal disease (ESRD) and may be due to an increase of anorexigenic peptides (leptin, ghrelin) and proinflammatory cytokines [interleukin (IL)-1, IL-8, tumour necrosis factor (TNF)-α] and a decrease of orexigenic hypothalamic neuropeptides (neuropeptide Y, agouti-related peptide) [8, 12–14]. This imbalance frequently causes reduction in energy and protein intake. Moreover, cachexia of dialysed patients arises as a result of a complex interplay of acidosis, a decreased response to anabolic hormones [insulin, insulin-like growth factor-1 (IGF-1)] and chronic inflammation [7–9]. The mechanisms underlying the muscle-protein breakdown are activation of specific proteases, such as caspase-3, and the ubiquitin-proteasome system [10, 11, 15]. This new knowledge opens the way to the development of specific drugs aimed at regulating the sense of appetite or counteracting the molecular mechanisms involved in muscle proteolysis.

## Assessment of nutritional status

Careful assessment of nutritional status is essential to identify the initial signs of cachexia. However, there is still no agreement as to the appropriate frequency of such assessments, which clearly depends on the age of the child: according to the National Kidney Foundation Kidney Disease Outcomes Quality Initiative (NKF KDOQI) nutritional guidelines, the majority of nutritional parameters should be assessed at least monthly in children aged younger than 2 years and every 3–4 months in older children. The complexity of the pathogenesis and clinical picture of cachexia in children on PD means that many indices should be considered, which can be grouped into seven different areas of investigation [16–19].

### Clinical and physical examination

A detailed history can be very informative, as children on dialysis may be very different from each other in terms of individual risk factors for protein-calorie malnutrition such as age, duration of CKD and dialysis, primary renal disease with proteinuria or water and sodium losses, possible concomitant acute or chronic inflammatory diseases, recurrent episodes of peritonitis and various other comorbidities such as multiple malformation syndromes, obesity and cardiovascular disease. The children or their parents should be asked about whether there have been any changes in appetite, gastrointestinal problems such as nausea, vomiting, diarrhoea, constipation or (in infants) gagging, any swallowing difficulties or an inability to chew solids and any changes in energy levels and activity when playing or attending school. Physical observations should focus on the condition of hair, teeth, tongue, skin, nails and breath, the impression of preserved or wasted fat or protein mass, dehydration or oedema and blood pressure. Thin hair, yellowish teeth lacking in enamel, pale furrowed tongue with scattered papillae on the surface, thin pale skin with sometimes bronze complexion and smelly fishy breath are peculiar to severe cachexia. However, although important, these are all subjective and should be considered late indicators of cachexia.

### Anthropometry

Anthropometric indices such as weight (W), height or length (H), height velocity (HV), head circumference (HC) and body mass index (BMI) are widely used in clinical practice, but measuring skin-fold thicknesses and body diameters requires special consideration. Direct measurements of tricipital skin-fold thickness and arm circumference can be used to calculate midarm muscle circumference (MAMC), arm muscle area (AMA) and arm fat area (AFA) by means of the following formula:

$$ MAMC = C - {\left( {{\pi T} \mathord{\left/  {\vphantom {{\pi T} {10}}} \right.  \kern-\nulldelimiterspace} {10}} \right)};AMA = {{\left( {10C - \pi T} \right)}^{2} } \mathord{\left/  {\vphantom {{{\left( {10C - \pi T} \right)}^{2} } {4\pi }}} \right.  \kern-\nulldelimiterspace} {4\pi };A = {\left( {\pi  \mathord{\left/  {\vphantom {\pi  4}} \right.  \kern-\nulldelimiterspace} 4} \right)}{\left( {{10C} \mathord{\left/  {\vphantom {{10C} \pi }} \right.  \kern-\nulldelimiterspace} \pi } \right)}^{2} ;AFA = A - AMA, $$

where C is midarm circumference (cm), T tricipital skin-fold thickness (mm), and A area of the arm (mm^2^) [20]. The anthropometric results should be compared with the corresponding control values and expressed as standard deviation scores (SDS) according to the following formula:

$$ {\text{SDS}} = {{\left( { \times  -  \times _{i} } \right)}} \mathord{\left/  {\vphantom {{{\left( { \times  -  \times _{i} } \right)}} {{\text{SD}}_{i} }}} \right.  \kern-\nulldelimiterspace} {{\text{SD}}_{i} } $$

where x is the individual patient value, x_i_ the mean value of the normal reference population and SD_i_ the standard deviation for normal controls. Age- and gender-specific reference values are available for the main anthropometric variables [20–25], but normal values stratified for pubertal status and race are lacking, which can affect the interpretation of anthropometric data [19]. Moreover, the use of chronological age for standardisation of anthropometric variables in patients on PD is questionable, given the growth deficit often present in this population. Although not formally proven, some form of correction for height age would be advisable [19].

One major drawback of all anthropometric indices is their lack of sensitivity in detecting mild and early alterations in nutritional status, and the main limitations of the parameters derived from skin-fold thickness are their poor reproducibility and the high degree of interobserver variability, because minimal variations in the direct measurements lead to large differences in the derived parameters [26, 27]. The key recommendation is therefore that the measurements should be made by the same person and at regular intervals.

Various authors have proposed predictive equations for estimating fat-free mass and fat mass based on anthropometric measures also in paediatric patients [28, 29]. Unfortunately, inaccurate estimates of body composition both at the individual and group level compared with estimates from dual-energy X-ray absorptiometry (DEXA) and research methods have been reported with the use of these equations [30–32].

### Dietary interview and diary

As cachexia is frequently associated with reduced protein and energy intakes, dietary assessment is a key step when evaluating dialysed children: it may not be strictly necessary for a final diagnosis of cachexia, but it does allow the prompt recognition of a factor that can lead to progressive decline in nutritional status. It is for this reason that nutrient intake, as estimated by means of dietary recall or by calculating the protein catabolic rate (PCR) was included in the minimal nutritional assessment recommended by the KDOQI in 2000 [16]. However, the use of 24-h dietary recall is by definition highly affected by the ability of parents to remember food details and quantities correctly; furthermore, the inability to identify day-to-day variability and the frequent under- or overreporting of dietary intake make the method insufficiently accurate to provide useful information. A multiple-day food record is obviously more precise than a dietary interview but requires the cooperation of parents, who should be given detailed instructions by a dietician. The duration of dietary diaries is still a matter of debate, and the need to capture day-to-day variability in nutrient intake by prolonging it should be balanced against the risk of inaccurate reporting. A 3-day diary is usually adequate, but the optimal duration should be individualised on the basis of the compliance of parents and children. The collected information should include protein and calorie intake, as well as fluids, electrolytes, fats and carbohydrates.

### Biochemical parameters

Many biochemical parameters have been proposed as a means of evaluating nutritional status in children and adults on chronic PD, including visceral proteins (albumin, prealbumin, retinol-binding protein, transferrin), serum creatinine and creatinine kinetics, total and partial lymphocyte counts and standard biochemistry. The most frequently used parameter in paediatric patients is serum albumin. A survey of prevalence data from the six-state New England area found that more than one third of the children with ESRD undergoing chronic PD had serum albumin concentrations <2.9 g/dl [5]. Wong et al. studied 1,723 paediatric patients and found that each 1 g/dl difference in serum albumin at the start of dialysis was associated with a 54% higher risk of death, even after adjusting for the glomerular causes of the ESRD and other potentially confounding variables [2]. However, serum albumin may also be influenced by fluid overload, chronic inflammation, urinary losses and the hyperpermeable status of the peritoneal membrane. Furthermore, whether hypoalbuminemia is predictive of mortality regardless of inflammation is still debated: according to the Hemodialysis (HEMO) Study, albumin is independently associated with nutrition and inflammation [33]. According to Yeun et al., the acute-phase response (or the cause of it) is largely responsible for the effect of hypoalbuminemia on mortality in HD patients [34]. Other biochemical parameters that can indirectly reflect nutritional status are haemoglobin and serum creatinine that, like total protein and serum albumin, have been found to be significantly lower in children on PD with an abnormal anthropometry-bioimpedance analysis nutrition (ABN) score than in those with a normal ABN score [6]. Low serum haemoglobin and creatinine levels may therefore indicate the need for a thorough nutritional assessment.

### Protein metabolism

Nitrogen balance (i.e. the difference between nitrogen intake and nitrogen losses) is one of the most powerful indices of metabolic status in patients on chronic dialysis. However, assessment of nitrogen losses can be complicated, as it involves urinary, dialysate and faecal collections and requires specific nitrogen measurements. We therefore measured nitrogen losses in the dialysate, urine and faeces of 23 children on chronic PD and elaborated a mathematical model for estimating nitrogen losses on the basis of the results of easily measurable laboratory dialysate and urine indices:

$$
{\text{TNe}}\;{\left( {{\text{g}} \mathord{\left/
{\vphantom {{\text{g}} {{\text{day}}}}} \right.
\kern-\nulldelimiterspace} {{\text{day}}}} \right)} = 0.03 + 1.138\;{\text{UN}}\;{\text{urea}} + 0.99\;{\text{DN}}\;{\text{urea}} + 1.18\;{\text{BSA}} + 0.965\;{\text{DN}}\;{\text{protein}},
$$

where TNe is total estimated nitrogen losses, UN urine nitrogen, DN dialysate nitrogen and BSA body surface area [35]. One major limitation of nitrogen balance is that it only indicates the balance between intake and losses at a given time and therefore anabolic or catabolic tendencies rather than nutritional status. Although steady-state total nitrogen appearance is the standard means of indirectly assessing dietary protein intake, only urea nitrogen appearance (UNA) is usually readily available. In children on PD in clinically stable conditions, UNA can be calculated from the sum of dialysate urea nitrogen and urinary urea nitrogen; in clinically unstable patients, changes in blood urea nitrogen have to be added.

The PCR, which is also called protein nitrogen appearance (PNA), can be calculated from UNA using specific regression equations. It is a useful indicator of protein intake in patients in steady state and more precise than the dietary protein intake obtained from dietary recall. However, it is still unclear whether the best formula for obtaining PCR is the modified Borah equations suggested by the KDOQI guidelines or that proposed by Mendley [36]. A significant association between PNA and hospitalisation and mortality has been reported for adult patients on dialysis [37, 38]. No data are available for paediatric patients.

### Body mass and body composition

Many methods have been proposed for the assessment of body composition, including DEXA, isotope dilution techniques, total body nitrogen and densitometry. However, as most of them are technically difficult, expensive and lead to the risk of exposing children to radiation or invasive procedures, they cannot be used on a regular basis.

Bioelectric impedance analysis (BIA) is a noninvasive, not-so-expensive and rapid means of assessing body composition at the bedside. Both single frequency and multifrequency analyses are widely used to assess the dry weight of children and adults on dialysis. The classic single-frequency method (distal and tetrapolar) provides two indices: resistance (R), which is considered to be an index of hydration, and reactance (Xc), which reflects both hydration and nutritional status. However, the interpretation of these results is still debated.

The first approach is to obtain lean body mass from BIA measurements. Many predictive equations have been proposed as a means of estimating body compartments on the basis of the R and Xc indices, but their use in clinical practice is not advisable [28] because they are based on assumptions that are not always true in children (such as constant hydration), and independent parameters such as height, weight and gender may account for as much as 80% of the result.

An alternative approach, vector BIA (BIVA), uses the R/Xc graph method: the R and Xc measurements are normalised by the stature of the subject, and vectors are plotted as points on the gender-specific 50th, 75th and 95th tolerance ellipses calculated from the healthy reference population. Changes in hydration are associated with movements of the impedance vector along a line of action lying parallel to the major axis of the reference tolerance ellipses, and alterations in nutritional status are associated with a shift that is parallel to the minor axis of the same ellipses [39]. The reference intervals of the whole-body impedance vector in Italian children have been determined by means of a cross-sectional, multicentre study [40]. However, although vector analysis allows an assumption-free assessment of tissue composition, more data is required concerning the paediatric dialysis population. Furthermore, BIVA is not quantitative and seems to be unsuitable for storing in a follow-up nutritional database.

A third approach consists of considering only the measured parameters (R and Xc) and the indices directly derived from them: phase angle (PA), which is calculated as the arc tangent of Xc/R, and distance (D), which is calculated as (PA × 10 + Xc)/2^1/2^. Reactance, phase angle and distance are all significantly lower in malnourished patients than in normal subjects and correlate well with other indices of nutritional status in children and adults. A number of studies of mainly adult patients have demonstrated a good correlation between BIA parameters and morbidity and mortality [41]. In a study of children treated with automated PD, we assessed anthropometric and BIA parameters during the first 24 months of dialysis and found that the BIA indices of Xc, PA and D were able to detect alterations in body composition earlier than anthropometry [42].

The best way to use BIA indices is to compare the measured values directly with previous data relating to the same patient, taking into account patient’s height increase, or with data from a reference population. This approach is very sensitive in identifying even subtle changes in body composition. BIA parameters may be influenced by body fluid content. In patients with an abnormal hydration status (such as children on PD), it is often difficult to distinguish whether the changes in bioelectrical values are due to alterations in the amount of water or the amount of body cell mass. However, there is no doubt that BIA is the most widely used method of assessing body composition in clinical practice worldwide and, with the above-mentioned caveats, is recognised as a useful tool for monitoring nutritional status in ESRD patients

### Nutritional scores

As no gold standard has yet been established, only the use of various assessments can facilitate more precise nutritional monitoring. The Subjective Global Assessment (SGA), which is proposed for adult patients on dialysis, is a widely used nutritional status scoring system based on medical history, physical examination and the physician’s grading of muscle wasting, subcutaneous fat and oedema; however, there is unfortunately no paediatric version [43, 44].

We recently proposed a nutritional score based on anthropometry and BIA. The ABN score uses nine noninvasive, inexpensive and reliable parameters (if measured by the same operator) that are easy to apply to both ill and healthy children: height, weight, BMI, MAMC, AMA, AFA, reactance, phase angle and distance [45]. All of these indices are expressed as SDS using a 5-point scale and then elaborated using a dedicated software programme to obtain an ABN score that may vary from 3 to 15. To establish the cutoff value between normal nutritional status and cachexia, it was first applied to healthy children, and distribution percentiles were calculated: an ABN score corresponding to the 3rd percentile (10.33) was established as the lowest limit of normality. In a large Italian multicentre cross-sectional study, 48.8% of the children on PD were found to have some degree of cachexia on the basis of their ABN score [6]. Not only all the parameters included in the score system but also serum albumin, haemoglobin and creatinine were significantly lower in children with an ABN scores of <10.33 than in those with an ABN scores of >10.33. The criteria used to select the nine ABN parameters, their application to a large healthy population and the first data coming from children on PD suggest that this scoring system may be useful in assessing the nutritional status of paediatric patients with ESRD. The major drawback of such a method is that it has been used so far in only one paediatric centre, and further studies are required to confirm the value of such observation.

In conclusion, although many indices have been proposed for the diagnosis of cachexia, no gold standard yet exists, and no single index can adequately reflect nutritional status [19]. Nutritional assessments should therefore be based on multiple methods whose results should be integrated by the paediatric nephrology team to make a comprehensive evaluation. According to the KDOQI guidelines, minimal nutritional assessments in adults on dialysis should include a visceral protein, such as serum albumin, a dietary interview or nPCR calculation, and an index of body composition [16]. The KDOQI guidelines adapted for children receiving maintenance dialysis include an estimate of nutrient intake by dietary interview, diary or PNA calculation, serum albumin and a complete anthropometric and growth assessment [16, 17, 19].

## Prevention and treatment of cachexia

Given the multifactorial etiopathogenesis of cachexia in children on PD, it should be approached in a multidisciplinary manner based on different steps [46].

### Dietary protein and calorie intake

The provision of adequate energy and protein intake is obviously fundamental, but anorexia may make it difficult in many cases. According to the KDOQI nutritional guidelines, the initially prescribed dietary energy intake for children on PD should be the recommended daily allowance (RDA) for their chronological age [16]. The calories derived from the glucose in the dialysate should also be considered when calculating daily total energy intake, because glucose absorption increases calorie intake by 7–10 kcal/kg per day [47]. Dietary prescriptions exceeding the RDA for age do not seem to be associated with any better outcomes in terms of albumin levels, morbidity or mortality and are currently not justified.

The dietary energy prescription should be adjusted on the basis of the child’s response. The KDOQI guidelines emphasise the importance of providing an adequate amount of nonprotein calories [16]: in a study of 31 dialysed children, the height velocity SDS correlated positively with total energy intake and negatively with daily protein intake [48]. Similarly, Azocar et al. found that a high protein intake seems to have a negative effect on acid-base status, bone mineralisation and fat-free mass [49].

As far as adequate dietary protein intake (DPI) is concerned, the possible losses of nitrogenous compounds in the dialysate should be taken into account [35, 50]. Albumin accounts for the majority of protein losses, and free amino acids for about 20% of total losses; but other proteins are also found in the dialysate, including immunoglobulins, transferrin and opsonin. The amount of protein loss is greatly affected by a series of factors, including peritoneal membrane permeability and peritoneal surface area, with proportionately higher losses being experienced by younger (and therefore smaller) children [50]. Protein losses of 100–300 mg/kg per day (approximately 10% of DPI) have been reported in children on PD, regardless of the treatment modality [50]. Episodes of peritonitis can lead to much greater protein and amino acid losses.

Nitrogen balance has been used as a means of determining adequate protein intake. We found that school-aged children on PD require a DPI of 144% RDA to obtain an estimated N balance of >50 mg/kg per day, which is considered necessary for growth [51]. Table 1 shows energy and protein intake levels recommended by the KDOQI Clinical Practice Guidelines for children undergoing chronic dialysis who are in stable clinical condition [16].

**Table 1:** Recommended energy and protein intakes for children on peritoneal dialysis (PD) according to the National Kidney Foundation Kidney Disease Outcomes Quality Initiative (NKF KDOQI) guidelines [16]

	Age (years)	Estimated energy allowances (kcal/kg per day)	Recommended dietary protein intake (g/kg per day)
Males and females	0–0.5	108	2.9–3.0
	0.5–1	98	2.3–2.4
	1–3	102	1.9–2.0
	4–6	90	1.9–2.0
	7–10	70	1.7–1.8
Males	11–14	55	1.7–1.8
	15–18	45	1.4–1.5
	18–21	40	1.3
Females	11–14	47	1.7–1.8
	15–18	40	1.4–1.5
	18–21	38	1.3

It is well known that chronic uraemia predisposes patients to a maladaptive state of low protein turnover that may be associated with a blunted response to high metabolic needs. In the case of accelerated protein degradation due to increased metabolic needs (for example, induced by an acute illness or conditions of stress), compensatory mechanisms such as increased protein synthesis may not be adequate. It is therefore particularly important to adjust calorie and protein provision during such periods to compensate for the increased protein degradation.

If spontaneous oral intake is inadequate, specific nutritional intervention should be considered. The first dietetic intervention is generally oral nutrient supplementation. Modular carbohydrate, fat and protein components can be used in infancy to increase the caloric density of the formula; in older children, energy and protein supplementation can be provided by modular components or commercial enteral products in liquid or bar form.

Enteral nasogastric, transpyloric or gastrostomy tube feeding should be considered in patients who fail to meet their nutritional goals by the oral route alone. There are conflicting results regarding the effectiveness of enteral supplementation in improving growth and nutrition. Most studies have reported an increase of height and mainly weight during enteral nutrition [52–54]. Conley et al. reported catch-up growth in 5/10 dialysed infants tube fed with 156–210% of the protein RDA and 108–134% of the energy RDA [52]. Ledermann et al. used a 30-month enteral feeding program to treat 35 children with chronic renal failure/ESRD (six on peritoneal dialysis) and concluded that long-term enteral feeding prevents or reverses weight loss and growth retardation, with significant catch-up growth if started before the age of 2 years [53]. Ramage et al. used gastrostomy feeding to treat 15 infants and older children on PD and concluded that it facilitates weight gain and arrests the decline in height SDS that is usually observed in infants with ESRD [54]. On the contrary, the results of a North American Pediatric Renal Trials and Collaborative Studies (NAPRTCS) survey of the effectiveness of supplemental feeding in 174 children aged younger than 6 years at the start of dialysis found no differences in weight or height SDS after 30 days, 6 months or 1 year of dialysis between the children who received supplementary feeds and those who did not [55]. However, limitations of registry studies should be taken into account when interpreting such data.

When comparing the different techniques of enteral nutrition, it is necessary to consider the advantages and limitations of each one. Nasogastric tubes are well tolerated and easy to insert, but their use may be complicated by recurrent episodes of emesis and the need for frequent tube replacement. Gastrostomy tubes or buttons are hidden beneath clothing but are also associated with emesis, exit-site infections, leakage and peritonitis [56]. Gastrojejunostomy should be limited to children undergoing enteral tube feeding when severe gastroesophageal reflux is not resolved by medical therapy. Percutaneous endoscopic gastrostomy (PEG) insertion following PD initiation carries a high risk for fungal peritonitis and potential PD failure. In such cases, open placement should be considered [57, 58]. Parents should be reassured that transition from a gastrostomy button to oral feeding following renal transplantation is generally successful without any significant delay.

### Intraperitoneal nutritional supplementation

Intraperitoneal amino acid supplementation is another option for children on PD, although it has been used in only a few paediatric patients and for short periods of time [59–61]. A Canadian randomised crossover study treated seven children for 3 months each with an amino-acid-supplemented dialysis solution and a standard dextrose solution and found no statistically significant between-period differences in terms of anthropometric parameters, total body nitrogen or questionnaire-based appetite scores [62]. Brem et al. reported the case of a 5-year-old PD patient treated for 1 year with a balanced 1.1% amino acid solution, whose serum albumin levels normalised and who showed significant increases in appetite, weight and linear growth velocity [63]. Some benefits in nutritional status, albumin levels and survival have been reported in adults on PD receiving intraperitoneal amino acids [64, 65]. Based on these experiences, it seems advisable to give PD-treated children with severe malnutrition a trial with an amino-acid-based PD solution together with the other nutritional measures. However, more studies are needed to better clarify the role of intraperitoneal amino acid supplementation in improving the nutritional status of children on PD.

### Pharmacological intervention

The use of dietary and pharmacological interventions to correct metabolic abnormalities (particularly acidosis, electrolyte abnormalities, anaemia and hyperparathyroidism) and treat complications such as infections and hypertension is very important in preventing and treating cachexia. Given the important role of drugs in controlling the blood chemistry of children with ESRD and the complications of uraemia mentioned above, particular monitoring is required to ensure the correct administration and timing of medications. Noncompliance with drug prescription is a common problem in chronic dialysis patients, particularly adolescents, and must be tackled by a collaborative effort of the multidisciplinary team [66]. The side effects of many drugs on the gastrointestinal tract should be taken into account, as they may negatively influence oral food intake.

Recombinant human growth hormone (rhGH) is one option to increase height velocity in children with CKD, being utilised in about 20–30% of children on dialysis, but its role in preserving lean body mass and improving whole-body protein homeostasis is not known in detail. Stefanidis showed an improvement in nitrogen balance and a significant increase in serum creatinine and creatinine excretion (with stable weekly creatinine clearance) in nine children on PD treated with rhGH, as well as an improvement in MAMC, the pattern of plasma amino acids and serum albumin [67]. A small number of studies of uremic adults suggest that rhGH is associated with a strong and sustained anabolic reaction and has a beneficial effect on nutritional status. On the basis of these data, the use of rhGH is advisable in selected children on PD with cachexia with the aim of improving not only growth but also nutritional status.

As regards other specific drugs for the treatment of protein-calorie malnutrition, megestrol acetate is the most extensively studied appetite stimulant. This drug has been shown to improve appetite and nutritional status in adults on hemodialysis, but the treatment may be risky and must be closely monitored [68, 69]. Efficacy and safety of nandrolone decanoate, an androgen derivative, have also been investigated in adults with CKD. Although an anabolic effect on lean body mass has been demonstrated, more studies are needed to confirm such observation [70]. To the best of our knowledge, no studies tested these drugs in paediatrics.

Given the association between proinflammatory cytokines and cachexia in patients on dialysis, various anti-inflammatory strategies have been proposed. It is worth noting, for example, that some drugs commonly used in patients with CKD, such as statins and angiotensin-converting enzyme inhibitors, possess an anti-inflammatory effect.

Given our ever-increasing knowledge about the pathogenesis of cachexia in patients with CKD, it can be expected that new drugs will be designed with the aim of specifically counteracting the molecular abnormalities (hormones, cytokines, neuropeptides) involved in nutritional status impairment. New and promising therapeutic interventions could be subcutaneous ghrelin administration and melanocortin receptor antagonists.

### Dialysis dose and maintenance of residual renal function

A close relationship has been found between dialysis dose and nutritional status, and that dialysis prescription (dialysis dose, dialysis solutions, ultrafiltration) and residual renal function should be monitored at least monthly. Peritoneal clearance should be regularly assessed by means of 24-h fluid collections and peritoneal membrane function by means of the peritoneal equilibration test. Kt/V and/or creatinine clearance data should be compared with the dialysis dose requirements laid down in the KDOQI guidelines [71], but they should only be used as indicators of prescription adequacy, because other clinical aspects may be more informative, such as control of uremic complications and growth itself [72]. The possibility of improving nutritional status by increasing the dialysis dose is still being debated, as there have been conflicting results concerning the relationship between the parameters of dialysis adequacy and nutritional indices in both adult and paediatric patients. The very definition of optimal dialysis is still controversial, but what is certain is that a PD dose of at least that recommended by the KDOQI guidelines is needed [73]. On the basis of these findings, the search for optimal dialysis dosing and nutritional intake is warranted.

## Conclusions

Despite recent advances in our knowledge of the mechanisms causing cachexia, both its early diagnosis and optimal treatment remain an open challenge. The prompt identification of cases of mild nutritional impairment requires the frequent monitoring of all of the physical, biochemical and instrumental signs of cachexia. Without a comprehensive evaluation of all indices, any conclusion about the nutritional status of a patient may be misleading.

As regards the treatment of malnutrition, further studies are needed to clarify the molecular abnormalities involved and determine specific treatment strategies. In the meantime, multidisciplinary efforts by physicians, dieticians, psychologists, nurses and parents are needed in an attempt to optimise every aspect of care. Such a common effort alone could prevent children from losing muscle mass and protein stores and thus allow better growth, reduced morbidity and a better quality of life.

## Questions

(Answers appear following the reference list)

1. Which one of the following methods can be considered as a gold standard for the diagnosis of protein-calorie malnutrition in children on peritoneal dialysis?
  - Bioimpedance analysis
  - Dual-energy X-ray absorptiometry
  - Serum albumin
  - Total body potassium
  - None
2. Anorexia in children with ESRD is due to:
  - Decreased leptin levels
  - Increased neuropeptide-Y levels
  - Increased proinflammatory cytokines levels
  - a+b+c
3. The recommended dietary calorie intake for a 5-year-old child on peritoneal dialysis according to the NKF KDOQI guidelines should be:
  - 50 kcal/kg per day
  - 70 kcal/kg per day
  - 90 kcal/kg per day
  - 110 kcal/kg per day
  - 130 kcal/kg per day
